# Genome Sequence of *Yersinia similis* Y228^T^, a Member of the *Yersinia pseudotuberculosis* Complex

**DOI:** 10.1128/genomeA.00216-14

**Published:** 2014-03-27

**Authors:** Lisa D. Sprague, Heinrich Neubauer

**Affiliations:** Friedrich-Loeffler-Institut, Federal Research Institute for Animal Health, Institute of Bacterial Infections and Zoonoses, Jena, Germany

## Abstract

We report here on the genome sequence of *Yersinia similis* 228^T^ isolated in Germany. The genome has a size of 4.9 Mb and a G+C content of 47% and is predicted to contain 4,135 coding sequences. Annotation of the 60,687-bp extrachromosomal element predicted 67 coding sequences and a G+C content of 47.8%.

## GENOME ANNOUNCEMENT

Initially typed as *Yersinia pseudotuberculosis*, *Yersinia similis* was identified as a separate species on the basis of differences in DNA-DNA relatedness, the presence of a specific 16S rRNA gene sequence, and the inability to ferment melibiose (1). *Y. similis* expresses the *Y. pseudotuberculosis*-derived mitogen YPMb but lacks the virulence plasmid pYV and the high-pathogenicity island and is therefore believed to be apathogenic for humans (1, 2). To date, *Y. similis* strains have only been isolated from small mammals and the environment. *Y. similis* isolates may cause diagnostic problems, as they cannot be biochemically distinguished from *Y. pseudotuberculosis* by commercial test kits, such as the widely used API 20E (1, 2). *Y. similis* Y228^T^ serovar VI was isolated from a rabbit in Germany.

This paper announces the genome sequence of *Y. similis* Y228^T^. Genomic DNA was isolated from an overnight culture grown in LB medium with 1% glucose with a Qiagen Genomic-tip 100/Q and genomic DNA buffer set (Qiagen, Germany). DNA quality was examined by using both a NanoDrop spectrophotometer (Thermo Scientific, Germany) and a Qubit 2.0 fluorometer (Life Technologies, Germany). Whole-genome sequencing was performed on a Pacific Biosciences RS sequencer with SMRT Technology PacBio RS II (Pacific Biosciences, Menlo Park, CA) at GATC Biotech (Germany) using standard protocols according to the manufacturer’s instructions, which were followed throughout the sequencing process. *De novo* assembly was performed with SMRT portal version 2.1.0 (Daemon version 2.1.0, SMRT View version 2.1, SMRTpipe version 2.1.0; Pacific Biosciences) from five contigs, with an *N*_50_ contig length of 4,779,767 bp. The genome has a size of 4,903,722 bp and a G+C content of 47%. Genome annotation was done using the NCBI Prokaryotic Genome Annotation Pipeline (http://www.ncbi.nlm.nih.gov/genome/annotation_prok/) and predicted 4,356 genes, 111 pseudogenes, 4,135 coding sequences (CDSs), and 86 tRNA and 22 rRNA genes. By using BLASTn 2.2.29 (3), a 60,687-bp extrachromosomal element with a G+C content of 47.8% was aligned to pGTD4 of *Y. pseudotuberculosis* (4), demonstrating the presence of a plasmid; its annotation predicted 67 CDSs.

The availability of the sequence of *Y. similis* 228^T^ may help to enlighten the evolution of apathogenic *Yersinia* to highly pathogenic *Yersinia* within the *Y. pseudotuberculosis* complex.

### Nucleotide sequence accession numbers.

The sequences of *Y. similis* 228^T^ and the putative plasmid of *Y. similis* 228^T^ have been deposited at DDBJ/EMBL/GenBank under the accession no. CP007230 and CP007231, respectively.
